# The Sick and Wounded in War: International Law and Usage

**Published:** 1915-01-09

**Authors:** 


### THE SICK AND WOUNDED IN WAR.

### International Law and Usage.

It is, of course, unnecessary to refer to a belligerent's treatment of his own sick and wounded, as it may be safely assumed that he will only be too anxious to care for and preserve them. The obligations of belligerents with regard to the sick and wounded of the enemy are governed by the Geneva Conventions of 1864 and 1906, the latter having practically supplanted the former. The Convention of 1864 agreed that as a compliment to Switzerland the emblem of the Red Cross should be retained as the distinctive sign and emblem of the sanitary services of hostile armies, and now the sign has universal significance. The Red Cross flag, however, is only to be hoisted with the consent of the military authority, and is to be accompanied by the national flag of the belligerent to which it belongs. It can only be used for the purpose of indicating the medical formations and establishments which are protected by the Conventions referred to. If neutral countries send Red Cross parties to the scene of action, their sanitary formations should fly the national flag of the belligerent to which they are attached along with the Red Cross flag. What international law exists on the subject of the care of the sick and wounded in war time is practically all contained in the Geneva Conventions, and may be summarised under the following headings:—

### (1) Treatment of the Sick and Wounded of the Enemy.

The soldiery and other persons officially attached to armies who are sick and wounded should be respected and cared for by the belligerent in whose power they are, without distinction of nationality; and a belligerent who is compelled to abandon sick or wounded to the enemy ought, as far as military exigencies permit, to leave with them a portion of his sanitary *personnel* and material to aid in caring for them.

After each engagement the belligerent in possession of the field of battle should take measures to search for the wounded and to ensure the protection both of the wounded and the dead against pillage and ill-treatment, and should also see that a careful examination of the bodies is made before the dead are buried or cremated, and as soon as possible send to the authorities of their country or army the military marks or tokens of identity found on the dead and a list of the names of the sick or wounded collected by him. Besides, the belligerents ought to keep each other mutually informed of any internments and transfers, as also of admissions into hospitals and deaths among the sick and wounded in their hands. They should collect all the articles of personal use, valuables, letters, etc., found on the field of battle or left by the sick or wounded who have died in the sanitary establishments or formations, in order that such articles may be transmitted to the persons interested by the authorities of their country.

### (2) Sanitary Establishments.

Movable sanitary formations, which are intended to accompany armies in the field, and the fixed establishments belonging to the sanitary service, must be respected and protected by the belligerents, but this protection ceases if they are used to commit acts injurious to the enemy. The following acts are not considered to be of such a nature as to deprive a sanitary establishment of protection:—

(1) That the *personnel* of the formation or establishment is armed and that it uses its arms for its own defence or for that of its sick and wounded.

(2) That in default of armed hospital attendants the formation or establishment is guarded by an armed detachment or by sentinels duly authorised.

(3) That arms or cartridges taken from the wounded and not yet handed over to the proper authority are found in the establishment.

### (3) Personnel.

The *personnel* engaged in the collection, transportation, and treatment of the sick and wounded, as also in the administration of the sanitary formations and establishments, are protected under all circumstances. If they fall into the hands of the enemy they are not treated as prisoners of war. The *personnel* of Voluntary Aid Societies, duly recognised and authorised by their Government, who are employed in the sanitary formations and establishments of armies, is assimilated to the regular *personnel*, provided that each State makes known to the other, either in time of peace or at the commencement of or during the course of hostilities—but in any case before actually employing them—the names of the Societies which it has authorised under its responsibility to render assistance to the regular sanitary service of its armies.

A recognised Society of a neutral country can only afford the assistance of its sanitary *personnel* and formations to a belligerent with the previous consent of its own Government and the authorisation of the belligerent concerned. The belligerent who has accepted such assistance is bound to notify his enemy before making any use thereof.

### (4) Material.

If movable sanitary formations, such as ambulances, fall into the hands of the enemy, they are allowed to retain their material. Nevertheless, the competent military authority shall have the right to use the material for the care of the sick and wounded. The buildings and material of fixed establishments remain subject to the laws of war, but they may not be diverted from their purpose so long as they are necessary for the sick and wounded. Nevertheless, the commanders of troops in the field may dispose of them in case of urgent military necessity, provided they make previous arrangements for the welfare of the sick and wounded who are found there. The material of Voluntary Aid Societies is considered private pro perty.

